# Supplementary material for: Analysis of PPARγ Signaling Activity in Psoriasis
Source: Int J Mol Sci. 2021 Aug 10;22(16):8603. doi: 10.3390/ijms22168603 (PMC8395241; doi:10.3390/ijms22168603)
Supplement: Supplementary file 1 [file ijms-22-08603-s001.zip › Supplemental materials_Analysis of PPARg signaling activity in psoriasis/Pathway models/Models images and html files/PPARG inhibits phenotypes assotiated with psoriasis/133635.html]

epithelial hyperplasia


# Disease epithelial hyperplasia

|  |  |
| --- | --- |
| URN | urn:agi-disease:epithelium%20hyperplasia |
| Total Entities | 0 |
| Connectivity | 573 |
| Name | epithelial hyperplasia |

---

|  |  |
| --- | --- |
| Pathway | PPARG inhibits phenotypes assotiated with psoriasis |
|  | efects selected |

---

|  |  |
| --- | --- |
| MedScan ID | 9602483 |

---

|  |  |
| --- | --- |
| Alias | epithelium hyperplasias |
|  | epithelium hyperplasia |
|  | hyperplasia of the epithelium |
|  | epithelial hyperplasia |
|  | epithelial hyperplasias |

---
